# Problems with the Current Approach to the Dissemination of Computational Science Research and Its Implications for Research Integrity

**DOI:** 10.1007/s11538-018-0499-y

**Published:** 2018-10-15

**Authors:** David Gavaghan

**Affiliations:** Department of Computer Science, University of Oxford, Wolfson Building, Parks Road, Oxford, OX1 3QD UK

Computational methods are at the heart of all twenty-first century research, but the acceleration of the application of computational approaches to biomedical research is particularly striking. From the simulation of the behaviour of complex systems, through the design and automation of laboratory experiments, to the analysis of both small- and large-scale data, computational approaches, and the well-engineered software that underpins them, have proved to be capable of transforming biomedical research. In parallel, the growth of high-throughput technologies and continual innovation in hardware, imaging, sensing and monitoring, demands unprecedented levels of collaboration between computational and experimental scientists, to continue the transformation of biology and medicine from primarily descriptive to quantitative, predictive disciplines. As a result, biomedical research is dependent as never before on computational science methods and hence also on the instantiation of those methods in research software.^1^

Despite this central and rapidly growing importance, biomedical research scientists are rarely trained to develop their own well-engineered software, nor are they trained to understand what it takes for research software to be transformative. Instead, research software is typically developed with the primary goal of facilitating rapid publication of a research group’s most recent results in the scientific literature. It is not usually made available to the research community, or even to reviewers, and so is not (and cannot be) verified.^2^ Significant research time is lost (usually by Ph.D. students with no formal training in software development) in re-implementing already-existing software tools from (often inadequate) literature descriptions. Even if successful, the re-implemented software is again not released to the community, and the cycle repeats. The huge benefits of verification, extensibility, sustainability and hence confident reuse of software in accelerating the progress of global research are rarely considered,^3^ nor are the implications of the current approach for the (lack of) reproducibility of published research.^4^,^5^ Progress in biomedical science is thus impeded, with knock-on effects into clinical translation and knowledge transfer into industry.

This issue is not, of course, limited to the biomedical domain but applies across almost all areas of computational science. As a research community it is therefore vitally important that we confront and find ways to deal with what is now one of the key issues in scientific research—that is, what is the most appropriate way to disseminate computational research^6^?

This question is particularly pressing in systems level science. No one group could ever hope to build a computational model of a complete biological or ecological system, and so progress is crucially dependent on being able to trust and then build upon the work of other researchers; genuine progress requires a collective endeavour on a par with that achieved in, for example, particle physics. However, all of the current drivers of research activity (bibliometrics, “alt”-metrics, funding mechanisms, the Research Evaluation Framework in the UK, typical metrics to qualify for tenure, the current academic publishing model, etc.) favour an individualistic approach, and so progress in systems research is painfully slow.

These issues have major implications for research integrity in the broadest sense, and in particular for the ability of the research community to assess the likely reproducibility of research, and to build on the research of others to accelerate the rate of scientific progress. Since the means of dissemination of research is at the heart of the problem, these issues also have major implications for the academic publishing industry.

## What Should We Do?

For younger researchers in Universities who are at the start of their academic careers, these issues throw up a dichotomy; that is, how to address complex research questions in the computational life sciences which require a collective approach, whilst at the same time scoring highly enough against the current metrics of academic “success” to be appointed to a tenured academic post? This dichotomy is apparent more generally across all interdisciplinary areas of science where research problems at the systems level are being tackled, and where progress is crucially dependent on the collective effort of the whole research community. The figure below and the questions placed alongside it attempt to capture this dichotomy.
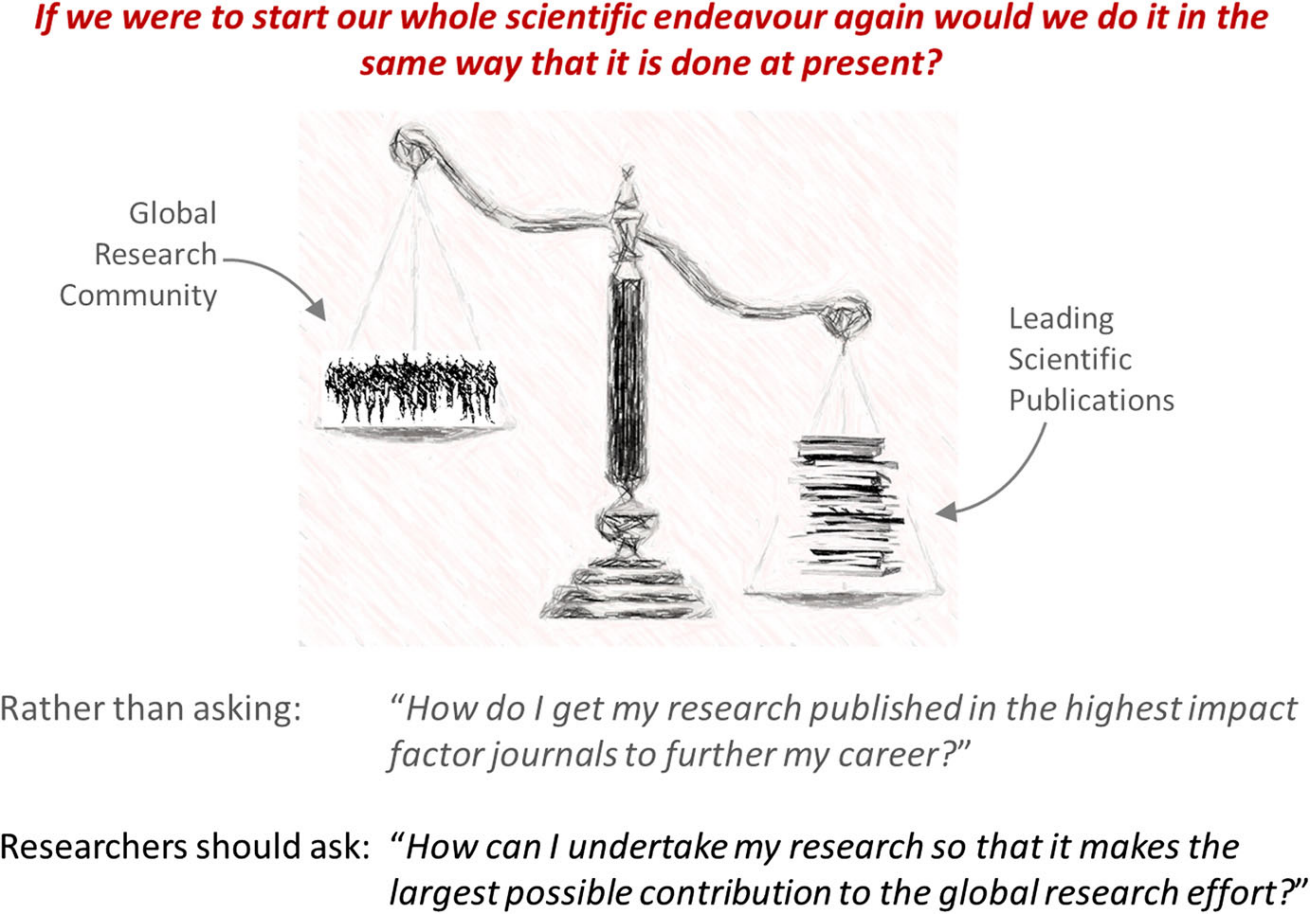


The alternative approach proposed in the figure of undertaking research so that it contributes optimally to the global research endeavour in a particular field, perhaps sounds naïve. However, this approach resonates very strongly with younger researchers, and the evidence is becoming overwhelming that the current approach to research dissemination is failing. If we accept that this new approach is indeed preferable, the natural question to ask is *“what are the most appropriate means of research dissemination to allow researchers to take this more collective approach?”*

This is the central question that we have been attempting to tackle in some areas of own research, and in particular in the recently completed 2020 Science research programme that was funded by the EPSRC (see the project website at http://www.2020science.net). The goal of this programme was to provide support and training to postdoctoral scientists to enable them to apply novel computational approaches to fundamental problems in natural science. Examples of the types of research questions addressed within the programme illustrate well the changing requirements for the dissemination of research in the natural and biomedical sciences at the system level:

- *Ecology* What is the best way to integrate models and data to obtain optimal information about the way in which species are currently distributed across the globe, and the way in which this distribution is changing in the face of changing climatic conditions?
- *Cardiovascular research* How do we make use of the over 100 computational models of cardiomyocytes developed over the last 50 years to assess the likely cardiotoxicity of potential new drugs earlier in the drug development pipeline?
- *Vision research* How can the eye adjust so rapidly to changes in light intensity?
- *Systems biology and medicine* Is there a rigorous mathematical and computational approach to moving from individual-based models to continuum-level population-based models?
- *Computational structural biology* How can we make state-of-the-art computational modelling tools accessible to the wider structural biology community?

These are precisely the types of systems level questions that cannot be addressed by an individual researcher (or research laboratory) in isolation; they are by nature collaborative and interdisciplinary. For these types of research questions, the standard approach to the dissemination of scientific research through journal publications and conference proceedings is one of the major obstacles to genuine community-level progress, and new approaches must be found.

In trying to support the researchers funded on the 2020 programme in developing new ways both to undertake and disseminate their research, our thinking evolved from early efforts which explored the feasibility of developing an overarching computational framework to support a wide range of computational science problems (our conclusion was that it cannot), to a more technologically heterogeneous and area-specific approach. This alternative approach perhaps mirrors that taken in other scientific areas, such as particle physics and genetics, where collective efforts have yielded spectacular successes. The approach that we are now taking, which we call the “online community resources” approach, evolved from our experiments with the use of executable papers, where we quickly realised that single executable versions of each paper merely exacerbates the problem of reusing other researchers’ efforts.^7^ This led us to ask ourselves three questions:

**Table:** 

1.	*“If I were a new computational researcher (e.g., a Ph.D. student or early postdoc) coming into this field, or an experienced researcher looking at this research for the first time, what would I need to have available to reproduce (or at least replicate) this research and then to build on it?”*

and in more mature areas

**Table:** 

2.	*“How can I provide (straightforward) access to my computational models to experimental/field scientists who want to make use of them in a way that promotes trust in the output, and ease of (re)use?”*

and finally (and most importantly)

**Table:** 

3.	*“How do we begin to generate communities of researchers who share a common research goal, and where computational models and experimental/field data are shared routinely in collectively addressing systems level research questions?”*

Underpinning these questions is our assumption that progress in systems level science needs a new approach to the dissemination of research outputs. Evidence of this need is clear in the recent plethora of publications highlighting the inability of the global research community to reproduce (often major) scientific results^3,4^. As suggested above, even in computational science, where at least the repetition of research should be straightforward, the underpinning software (which is the instantiation of the mathematical and computational models) is typically not made available to the research community and therefore cannot be verified. Vast amounts of research time is lost in developing new software to re-implement existing models before those models can first be verified prior to any consideration of reuse and extension. Given that in the UK alone, research councils are estimated to be spending in excess of £800 m a year^8^ in projects developing and using research software, this approach is unacceptable.

Over the last two years of the 2020 project, we worked with the fellows to identify and support areas of their current research programmes that might provide compelling examples of the utility of a web-based community approach. The examples that we developed reached varying stages of maturity. The project that has gained the most traction, the Cardiac Weblab^9^ (see https://travis.cs.ox.ac.uk/FunctionalCuration/), pre-existed the 2020 project and has received follow-on funding to continue to build the online resource and to connect with the relevant research community. This project had the strong advantage of being able to build on a widely adopted XML-based model description language and repository (CellML—see https://www.cellml.org/). A second project on species distribution modelling^10^ (see https://zoonproject.wordpress.com/) has gained reasonable traction in the relevant community but requires further funding to meet its original goals. This project grew from initial discussions with the Weblab team and again developed a standard-based approach to species distribution modelling, in this case a workflow standard in the R programming language. This highlights two further issues in the quest to improve the reproducibility of computational science research: firstly, the need for machine-readable agreed standards for describing the computational model since this is a prerequisite for routine and straightforward model checking and reuse by the wider community; and secondly (as discussed briefly in the next section), the long-term sustainability of such online resources once the initial funding stream has ended, and the implications of this issue for the academic publishing industry.

In attempting to address the three questions posed in the boxes above, these online community resources are designed (ultimately) to become the “first stop” for researchers wishing to discover the state of the art in computational approaches in a particular research domain. The intention is that all of the resources necessary to allow researchers to make an informed choice of the best option from competing models and software will be available in a single location. As a first (essential) step, in our own research, we now make available all of the information (the “research assets”) necessary to repeat the results in our own publications (data, models, software, data analysis and visualisation tools, tutorials and exemplars). The next step, which has commenced in the Weblab project, is to build the infrastructure necessary to allow individual researchers and groups in the wider community to upload their own software, models and data and compare them against both our and other competing computational models, algorithms and approaches.^11^

Our intention is that researchers will also be able to “validate” their approach against any experimental data that we can make available or that we can link to.

This approach moves well beyond simply providing open access to the source codes of the software that has been used in deriving the results included in published papers. Although such open access is the most basic requirement for improving the repeatability (and reproducibility) of computational modelling work, our approach also opens up the critical requirements of building community-wide trust in the implementation and output of computational models and paves the way for the routine reuse of both computational models and of the experimental data on which those models are based. Our goal is that these online resources will support the necessary collective approach within each research community, in both the development, reuse and extension of computational models (and software), and in the use and curation of the experimental and field data used to derive and test those computational models. Data and models will then be freely available to all within each community, allowing those communities to begin to address common research goals in systems level research, as advocated in the three questions that we have posed above.

## What is the Role of the Academic Publishing Industry?

The issues raised above concerning the (lack of) reproducibility of research have major implications for the academic publishing industry. If a significant proportion of the key scientific research findings underpinning a discipline proves to be non-reproducible,^12^,^13^ this has profound implications for the current journal-based and peer-reviewed publications process—we simply must find a more robust way. Much of the problem is due to the rapid acceleration of academic publication over the last two decades—researchers are under pressure to publish more and to publish it faster—and the current review system cannot cope. Some publishers are now turning to a more collective reviewing system (for example, *Wellcome Open Research*, *eLIFE* and the *Frontiers* journals), and this is part of the solution. However, this does not address the issue of community *ownership* and *custodianship* which I believe is crucial to addressing research reproducibility. Academic publishers already have the building blocks in place to provide this support through their information management platforms and their existing communities which are typically aggregated around particular specialist journals, with community leadership often provided by journal editorial boards. What is needed is a rethink of what constitutes a “journal”. As suggested above, journals of the future will be information repositories that contain the outputs of research presented to its user base (the community which “owns” it) in a manner which makes it as easy as possible to replicate, reproduce, reuse and compare those outputs. There is still an essential role for peer review, although the review process will need to evolve as an ongoing activity that ensures that the community can make fully informed judgements on the current state of knowledge in particular areas and can critically assess new findings with full access to all of the necessary information. Academic publishing in the future then becomes the provision of the information platform to support the work of a particular community, effectively becoming part of that community. The role of the academic publisher within the community is then to act as the initial gatekeeper to the platform to ensure that the research has been done responsibly using appropriate research methods, and then to foster and support that community as it acts (collectively) to move its discipline forward in a manner designed to ensure the highest degree of (collective) utility in the research being undertaken. In the particular case of computational science research, academic publishers will also have a key role in promoting the development and use of standard-based approaches to research dissemination, whether those standards are to support model-sharing, data-sharing or well-engineered software. This, in turn, will help to foster the highest possible levels of research integrity.

## Footnotes

1 A recent survey by the national Software Sustainability Institute in Edinburgh found that 96% of biomedical researchers use research software, and 69% believe their research would be impractical without it. Hettrick et al., 2014. 10.5281/zenodo.14809.

2 Notably, this issue is highlighted in the March 6th 2018 *Nature* Editorial, see https://www.nature.com/articles/d41586-018-02741-4.

3 Ince DC, Hatton L and Graham-Cumming J. The case for open computer programs, Nature, 482, 485–488, 2012, https://www.nature.com/articles/nature10836.

4 Stodden V, Seiler J, Ma Z. Journal policy for computational reproducibility. Proceedings of the National Academy of Sciences. 2018, 115, 2584-2589. 10.1073/pnas.1708290115.

5 Peng RD. Reproducible research in computational science. Science. 2011, 334:1226– 1227. 10.1126/science.1213847.

6 Note that computational research here should be taken to have a broad reach and includes any research which is reliant on software, and so includes data science techniques.

7 Whilst an individual executable paper typically allows the reader to explore the data and results embedded in the paper in more detail, we quickly realised that this does not improve the ability of the reader to build on the findings. In order to do this, the *research assets* underpinning the paper and that would make it reusable (e.g., data, models, software, data analysis and visualisation tools, tutorials and exemplars) must also be made available to the research community.

8 http://www.software.ac.uk/blog/2014-10-22-840-million-uks-investment-software-reliant-research-2013.

9 Cooper J, Mirams GR, Niederer SA, High-throughput functional curation of cellular electrophysiology models, Progress in Biophysics and Molecular Biology, 2011: 107; 11–20. 10.1016/j.pbiomolbio.2011.06.003.

10 Golding N, August TA, Lucas TCD, Gavaghan DJ, van Loon EE, McInerny G. The Zoon R package for reproducible and shareable species distribution modelling. *Methods Ecol Evol*. 2018;9:260–268. 10.1111/2041-210X.12858.

11 Daly AC, Clerx M, Beattie KA, Cooper J, Gavaghan DJ, Mirams GR, Reproducible model development in the cardiac electrophysiology Web Lab, Progress in Biophysics and Molecular, Biology, 2018, *in press*, available at 10.1016/j.pbiomolbio.2018.05.011.

12 Prinz F, Schlange T and Asadullah K. Believe it or not: how much can we rely on published data on potential drug targets? Nature Reviews Drug Discovery, 10, 712 (2011). https://www.nature.com/articles/nrd3439-c1.pdf.

13 Begley CG and Ellis LM. Drug development: Raise standards for preclinical cancer research, Nature, 483, 531–533, 2012), https://www.nature.com/articles/483531a.pdf.

